# The sulfur assimilation pathway mitigates redox stress from acidic pH in *Salmonella* Typhi H58

**DOI:** 10.1128/mbio.00467-25

**Published:** 2025-05-27

**Authors:** Marion Fernandez, Yuki Yamanaka, Parisa Zangoui, Mark Andrew White, Linda J. Kenney

**Affiliations:** 1 Department of Biochemistry and Molecular Biology, University of Texas Medical Branch 198643, Galveston, Texas, USA; 2 Mechanobiology Institute, National University of Singapore 145756, Singapore, Singapore; 3 Sealy Center for Structural Biology, University of Texas Medical Branch, Galveston, Texas, USA; National Institutes of Health, Bethesda, Maryland, USA

**Keywords:** *Salmonella *Typhi, H58, redox, sulfur assimilation, SifA, SsrB, *Salmonella* pathogenicity island 2, acid stress response

## Abstract

*Salmonella enterica* serovar Typhi is the causative agent of typhoid fever, a human-restricted systemic infection. The rapidly disseminating multi-drug resistant H58 subclade is endemic in Africa, Asia, and Oceania, yet little is known regarding its intracellular behavior and virulence properties. It was of interest to understand the H58 response to host cell infection in terms of its response to acid stress and subsequent changes in gene regulation. We compared the H58 response *in vitro* and during infection of THP-1 human macrophages with the well-characterized response of *Salmonella* Typhimurium, which causes gastroenteritis. In *S*. Typhimurium infections, bacteria reside in an acidic intracellular vacuole and become acidified, driving the expression of pathogenicity island 2 genes (SPI-2). SPI-2 virulence factors modify the vacuole and enable bacterial replication. In response to acid stress, the sulfur assimilation pathway is highly upregulated and unique to H58. Replacing the Typhi *cysK* promoter with the Typhimurium promoter resulted in a *cysK* gene that was upregulated by acid stress in the H58 background, highlighting the differential regulation between the two serovars. In acidic conditions, H58 experienced much greater redox stress compared with *S*. Typhimurium, and the sulfur assimilation pathway was required to mitigate the redox stress. Higher redox stress modified the transcriptional regulator SsrB, resulting in diminished secretion of the SPI-2 virulence factor SifA. Our results highlight significant differences between *S*. Typhi H58 and *S*. Typhimurium and emphasize the importance of studying *S*. Typhi strains directly to understand their unique behavior during pathogenesis.

**IMPORTANCE:**

In this study, we examined the clinically relevant, multi-drug resistant *Salmonella* Typhi strain H58, which is rapidly disseminating across Southeast Asia, Africa, and Oceania. It has heretofore been uncharacterized in terms of its gene regulation. Using human THP-1 macrophages, we discovered that S. Typhi strongly activates the sulfur utilization pathway in response to acid stress encountered in the vacuole once Typhi is inside host cells. Our novel findings were that *S.* Typhi experiences substantially higher redox stress compared with Typhimurium, and it requires the sulfur utilization pathway to mitigate this stress. This pathway is not upregulated in Typhimurium and represents a divergence in the response of these two serovars. We emphasize that *S.* Typhimurium is not a reasonable model for understanding H58, a serovar that is seriously impacting human health.

## INTRODUCTION

*Salmonella enterica* serovar Typhi (*S*Ty) is the causative agent of typhoid fever, a human-restricted severe systemic infection. The global incidence of typhoid fever is between 12 and 27 million in mostly children, adolescents, and older adults. In total, 116,815 people succumbed to typhoid fever in 2017 (1). The global *S*Ty population is highly structured and includes dozens of subclades that display geographical restriction. The exception is the rapidly disseminating multi-drug resistant H58 subclade, now designated genotype 4.3.1, which is endemic in Africa, Asia, and Oceania (2). Effective combating of typhoid fever has forever been challenging, due to the rise of multi-drug-resistant H58 strains, weakly protective vaccines, and the long-term colonization of *S*Ty in chronically infected patients as biofilms (3–6). Typically, all patients infected with *S*Ty shed bacteria in the environment, as bacteria leave systemic sites, such as the gall bladder and re-enter the intestines to pass in the feces. In around 2%–4% of cases, *S*Ty infections do not resolve as they enter the persistent life cycle, leading to prolonged fecal shedding in the absence of any disease symptoms (7). Such seemingly healthy individuals become asymptomatic carriers, as the infamous case of “Typhoid” Mary Mallone in the early 1900s. In modern times, frequent movements of such human carriers from endemic regions, such as South America and Asia to non-endemic regions such as North America, have caused the majority of typhoid cases.

Because *S*Ty is human-restricted, *Salmonella enterica* serovar Typhimurium (*S*Tm) is often used as a surrogate in understanding *S*Ty, an assumption that we challenge herein. Although *S*Tm causes gastroenteritis in humans, it causes a systemic disease in the mouse (8–12). Our overall understanding of *Salmonella* as a pathogen from cell culture and mouse studies has produced the following general model of its pathogenesis. Upon ingestion of contaminated food or water, some bacteria survive the extreme acid pH (1.5–3.5) of the stomach and enter the intestine. *Salmonella* senses the host environment and activates expression of a type three secretion system (TTSS1) that is encoded on a pathogenicity island (*Salmonella* pathogenicity island 1 [SPI-1]) acquired during the evolution of *Salmonella* as a pathogen (9, 10, 13–15). SPI-1 and its associated virulence factors catalyze its uptake across the intestinal epithelium. Once inside cells, *Salmonella* resides in a vacuolar compartment termed the *Salmonella*-containing vacuole (SCV) (15, 16). In this compartment, *Salmonella* responds to the acid pH of the vacuole by intracellular acidification catalyzed by the EnvZ-OmpR two-component regulatory system (17–20). This leads to the upregulation of SsrA-B, a two-component system residing on pathogenicity island 2 (SPI-2) (17–23), producing TTSS2. In response to acid stress, the response regulator SsrB, through a conserved histidine residue in its phosphorylation domain (His12), drives a 30-fold change in DNA binding affinity of its C-terminus, which activates a second type three secretion system (23, 24). The TTSS2 on SPI-2 and its associated secreted effectors result in endosomal tubulation (25), a process referred to as *Salmonella*-induced filament (SIF) formation driven by the effector SifA (26, 27) and other interacting effectors. Additional SPI-2 virulence factors modify the vacuole, preventing its degradation and allowing *Salmonella* to replicate within this acidic compartment (28, 29).

In bacteria, the sulfur assimilation pathway catalyzes the synthesis of cysteine using sulfate and thiosulfate as precursors. The enzymes in charge of the diverse reactions are encoded by single genes or operons regulated by the master regulator CysB. CysB is known to respond to O-acetylserine and sulfur limitation to activate *cysJIH, cysK,* and *cysP*, as well as inhibiting itself (reviewed in reference 30). The sulfur assimilation pathway, along with cysteine, plays a major role in cellular redox balance. For instance, synthesis or degradation of cysteine directly impacts the pool of H_2_S within the cell, which is known for its antioxidant properties, depending on the concentration (31). In addition, cysteine is a precursor for the synthesis of glutathione, a low-molecular-weight thiol that assists in maintaining redox balance in cells (32, 33). Sources of oxidative stress can be exogenous (such as coming from host cells) or endogenous (e.g., bacterial metabolism), and maintaining the bacterial redox state is essential for bacteria to maintain proper functions. Oxidative stress can damage all types of cellular components (34), and it also influences gene expression in *Salmonella* (35, 36).

We were interested in understanding how H58, a rapidly disseminating multi-drug-resistant strain that has not been well characterized, responded to host cell infection in terms of its response to acid stress and its subsequent changes in gene regulation. We compared the H58 response with the well-characterized response of *S*Tm *in vitro* and during infection of THP-1 human macrophages. In response to acid stress, the sulfur assimilation pathway was highly upregulated in a process that was unique to H58 and not observed in *S*Tm. Measurements of the redox state of H58 in acidic conditions indicated that it experienced much greater redox stress compared with *S*Tm, and the sulfur assimilation pathway was required to mitigate the redox stress. Higher redox stress modified the transcriptional activity of the regulator SsrB, resulting in diminished secretion of the SPI-2 virulence factor SifA. Our results highlight important differences between *S*Ty H58 and *S*Tm and emphasize the need to study *S*. Typhi strains directly in order to understand their unique behavior during pathogenesis.

## MATERIALS AND METHODS

### Bacterial strains and culture conditions

*Salmonella enterica* serovar S. Typhi H58 lineage, ST02TY06, was obtained from Dr. Stephen Baker (Cambridge Infectious Disease, Cambridge, UK). pZS::P*tet*-mCherry and pFPV25::roGFP2 were kindly provided by Professor J. Ling (University of Maryland) and Professor Finlay (University of British Columbia), respectively. Strains used for this study are listed in Table 1. Bacterial cultures were grown in LB media supplemented with 100 µg/mL ampicillin, 15 µg/mL chloramphenicol, 50 µg/mL kanamycin, or 12.5 µg/mL tetracycline when required. The LB media had a pH_e_ of 7.0 or adjusted by HCl to pH_e_ 4.5, and cells were grown at 37°C with shaking at 250 rpm, or in standing conditions if mentioned. For SPI-1-inducing conditions used for cell infections, 3 mL of overnight standing culture was inoculated from a single colony. For SPI-2-inducing conditions, an overnight LB culture of *S*. Typhi was diluted in LB pHe 4.5 at a dilution of 1/30. Cultures were grown until the optical density at 600 nm (OD_600_) was 1.0 (~6 h).

**TABLE 1:** Strains and plasmids

Strain or plasmid	Description or genotype	Reference or source
*S*. Typhi strains		
H58	WT	Lab stock
∆*ssrB*	H58 ∆*ssrB*::Cm	Lab stock
∆*cysB*	H58 ∆cysB:Cm	This study
∆*ompR*	H58 ∆*ompR*::Cm	This study
∆*phoP*	H58 ∆*phoP*::Cm	This study
∆*cysK*	H58 ∆*cysK*::Cm	This study
*cysK+*	H58 ∆*cysK*::Cm, deletion complemented by chromosomal expression of *cysK* integrated via miniTn7	
∆*cysK ssrB*^C203A^	H58 ∆*cysK, ssrB* C203A mutation at the locus	This study
P*cysK^S^*^Ty^-mCherry	H58 pFPV::P*cysK^S^*^Ty^-mCherry. Plasmid expressing mCherry under the control of the PcysK amplified from STy H58	This study
P*cysK^S^*^Tm^-mCherry	H58 pFPV::P*cysK^S^*^Tm^-mCherry. Plasmid expressing mCherry under the control of the P*cysK* amplified from STm 14028s	This study
P*cysK^S^*^Ty^-mCherry-Tn_7_	H58 *att*Tn*7*:: P*cysK^S^*^Ty^-mCherry. Chromosomal expression of mCherry under the control of the P*cysK* amplified from STy H58. The strain also expresses GFP constitutively from pFPV25::*gfp*	This study
∆*omp*R P*cysK^S^*^Ty^-mCherry-Tn_7_	H58 ∆*ompR*:: Cm, *att*Tn*7*:: P*cysK^S^*^Ty^-mCherry. Chromosomal expression of mCherry under the control of the P*cysK* amplified from STy H58	This study
+*omp*R WT P*cysk*^STy^-mCherry-Tn_7_	H58 ∆*ompR*:: Cm, *att*Tn*7*:: P*cysK^S^*^Ty^-mCherry, pSKW::*ompR* Chromosomal expression of mCherry under the control of the P*cysK* amplified from STy H58. Complementation of *ompR* deletion from pWSK plasmid	This study
+*omp*R D56A P*cysk*^STy^-mCherry-Tn_7_	H58 ∆*ompR*:: Cm, *att*Tn*7*:: P*cysK^S^*^Ty^-mCherry, pSKW::*ompR* Chromosomal expression of mCherry under the control of the P*cysK* amplified from STy H58. Expression of *ompR* gene carrying D56A mutation from pSKW plasmid	This study
P*sifA-*mTagBFP2	H58 pZS:: P*sifA-*mTagBFP, P*tet*-mCherry. Expression of mTagBFP under the control of P*sifA*, constitutive expression of mCherry	This study
∆*cysK* P*sifA-*mTagBFP2	H58 *cysK*::cm. pZS:: P*sifA-*mTagBFP, P*tet*-mCherry. Expression of mTagBFP under the control of P*sifA*, constitutive expression of mCherry	This study
roGFP2	H58, pFPV25::roGFP2. Expression of the redox-sensitive roGFP2 mutant	This study
∆*cysK* roGFP2	H58, ∆*cysK*::Cm, pFPV25::roGFP2. Expression of the redox sensitive roGFP2 mutant	This study
*S*. Typhimurium strains		
14028s	WT	Lab stock
∆*cysK*	14028s ∆*cysK*::Cm	This study
∆*cysK* P*sifA-*mTagBFP	14028s ∆*cysK*::Cm. pZS:: P*sifA-*mTagBFP, P*tet*-mCherry Expression of mTagBFP under the control of P*sifA*, constitutive expression of mCherry	This study
Plasmids		
pKD3	Cm^R^	(37)
pKD46	Amp^R^	(37)
pWSK29	Low copy number cloning vector, *rep*^101^ Amp^R^	Lab stock
pFPV::P*cysK^STy^-mCherry*	Reporter for P*cysK* of H58 activity	This study
pFPV::P*cysK^STm^-mCherry*	Reporter for P*cysK* of H58 activity	This study
pWSK29::*ompR*	Amp^R^	Lab stock
pWSK29::*ompR*^D55A^	Amp^R^	Lab stock
pET15b-OmpR	Production and purification of OmpR, Amp^R^	(38)
pFPV25:: roGFP2	Amp^R^, Expression of the redox sensitive roGFP2 mutant	(39)
pZS::P*tet*-mCherry	*rep*^101^, Amp^R^	(40)
pZS::P*sifA*-mTagBFP2	*rep*^101^, Amp^R^, insertion of P*sifA*-mTagBFP into pZS::P*tet*-mCherry by Gibson assembly	This study
pTNS2	OriR6KT, helper plasmid for integration of miniTn7	Addgene #64968
pUC18R6KT-mini-Tn7T-Km	OriR6KT, Integration onto bacterial chromosome at *att*Tn*7* site	Addgene #64969
pTn7-*cysK*	pUC18R6KT-mini-Tn7T-Km carrying *cysK* genes from H58 for chromosomal integration	This study
pMPM_SsrBWT	pMPMA5Ω plasmid cloned with 6xhis-ssrB, under the control of arabinose promoter (Amp^R^)	(22)
pMPM_SsrB K179A	pMPM_SsrBWT with SsrB K19A mutation	Lab stock
pMPM_SsrB C203A	pMPM_SsrBWT with SsrB C203A mutation	Lab stock

### Mutant and plasmid construction

For the construction of *ompR*, *phoP*, *cysB*, and *cysK* null strains of *S*. Typhi or *S*. Typhimurium, genes of interest were replaced by the chloramphenicol cassette (CmR), amplified from pKD3 with primers listed in Table 2, using λ-Red recombination techniques (37). Mutants were selected on LB agar supplemented with 15 µg/mL chloramphenicol. Deletions were verified using internal and deletion flanking primers. pUC18R6KT-minT7-Kn was integrated into the bacterial chromosome with pTNS2 as a helper plasmid, and transformants were selected on LB agar supplemented with kanamycin 50 ng/mL (41).

**TABLE 2:** Primers used in this study

Purpose and primer	Sequences
Deletion by lambda Red, amplification of Cm cassette onto pKD3	
*ompR*	5′TGTTGCGAACCTTTGGGAGTACAGACAATGCAAGAGAATTATTAGGTGTAGGCTGGAGCT-3′ 5′GGCGAGAAGCGCATTCGCCTCATGCTTTAGAACCGTCCGGTACATGAATATCCTCCTTAG-3′
*phoP*	5′TCTTATTGTTAACACAAGGGAGAAGAGATGATGCGCGTACTGTAGGTGTAGGCTGGAGCT-3′ 5′GATATCCTTGTCCGCGTACGGTGGTAATGACATCGTGCGGATAATGAATATCCTCCTTAG-3′
*cysK*	5′GATAACTCGCTGACTATCGGTCATACGCCGCTGGTTCGACTGGTGTAGGCTGGAGCTGCTTC-3′ 5′GTTGCAGTTCTTTCTCAGTAAAGAGATCGGCGAACAACGCGGCATATGAATATCCTCCTTAG-3′
*cysB*	5′CGATTAAGTCTGCAGATAAACGATGGCCTGATGGCGCTAATCTGGGTGTAGGCTGGAGCT −3′ 5′GGCTTTGTCTGCCATGCCACTACGACACAAACCGACGGTGATAATGAATATCCTCCTTAG −3′
qPCR	
*cysB*	5′CGCCCGCAGTGGTAAGCATC-3′/5′GAGCCTTTATCCGGCCAGGT-3′
*sbp*	5′GCACCTTACACCTCCACCAT-3′/5′TAGTTCCAGCGTGCGCCGCC-3′
*cysK*	5′CCATGCCGGAAACCATGAGC-3′/5′GCGGATCGCTGGCGACAATT-3′
*rrsA*	5′gcaccggctaactccgtgcc-3′/5′gcagttcccaggttgagcccg-3′
EMSA	
P*cysK*	*cysK*-EMSA-F: 5′TTGGCGCGCTCACCAGCCTC-3′ *cysK*-biotin-R: 5′biotin-GGCCTGTCCTTAACTGTATG-3′ *cysK*-EMSA-R (unlabeled DNA): 5′GGCCTGTCCTTAACTGTATG- 3′
Cloning: Gibson assembly	
P*sifA*	5′TTATCAGAATCGCAGATCTGTTGATTTACTGGCGGGCT-3′ 5′CGCCCTTAGACACCATATTAATCTCACTTATACTGG-3′
mTagBFP2	5′CCAGTATAAGTGAGATTAATATGGTGTCTAAGGGCGAAGA-3′ 5′CAATTCTGGAAGAAATAGCGAAAGGCCGCTAAAGCGGCT-3′
pZS::Ptet-mCherry vector	5′AGCCCGCCAGTAAATCAACAGATCTGCGATTCTGATAAC-3′ 5′CGGCCTTTCGCTATTTCTTCCAGAATTG-3′
Cloning: enzymatic restriction	
*cysK* into pUC18R6KT	5′ATAGGTACCTTGGCGCGCTCACCAGCCTC-3′ (KpnI) 5′ATAGAGCTCCCGTTGCTGGCAACTTATGG-3′ (SacI)

Plasmids pFPV::P*cysK^STy^-mCherry* and pFPV::P*cysK^STm^-mCherry* and pTn7-P*prgH*-mCherry were made by Gibson assembly (NEB) according to the manufacturer’s instructions (Table 2). The construction of pZS::P*sifA*-mTagBFP2 was also made by Gibson Assembly. P*sifA* was fused to mTagBFP2, amplified from mTagBFP2, and integrated into pZS:: Ptet-mCherry. For the construction of pTn7-*cysK*, *cysK* was amplified, and the amplicon was digested with KpnI and SacI restriction enzymes for integration into pUC18R6KT-miniTn7-Kn.

### Mass spectrometry

An overnight culture of *S*. Typhi was grown as described above, and a 1 mL culture was pelleted and resuspended in TE buffer, pH 8.0, containing 1% (wt/vol) of SDS and protease inhibitor AEBSF, for 1 h at 37°C. Samples were then centrifuged at 130,000 × *g* for 15 min, and the supernatant was collected. The total protein concentration was assessed by the Bradford assay. The samples were then prepared as described (42). Briefly, 1 µg of protein was digested using the S-Trap (protifi.com) manufacturer’s recommended protocol. LCMS was performed using a trap and elute method on an Orbitrap Fusion, and resulting files were searched against the *S*. Typhi, and a contaminant cRAP formatted fasta databases using Sequest and Proteome Discoverer 2.5.

### RNA extraction, RNAseq, and qPCR

*S*. Typhi was grown in LB medium overnight and subcultured in LB medium pHe (4.5 or 7.0) until the OD_600_ reached ~1.0. The total RNA was isolated using a GeneAll Hybrid-RTM kit (GeneAll Biotechnology) for isolation of RNA from tissues and cultured cells, following the manufacturer’s instructions. Purified RNA was treated with DNase I using the TURBO DNA-freeTM kit from Ambion (Life Technologies). Rigorous DNase treatment was performed by incubation with 1 µL TURBO DNase (Invitrogen) according to manufacturer’s instruction. The DNase was inactivated with DNase inactivation reagent for 5 min. The quality of RNA was examined with Bioanalyzer 2100 (Agilent Technologies). RNA samples with RNA integrity number (RIN) >6 were processed for sequencing by NovogeneAIT Genomics, Singapore. Depletion of ribosomal RNA and preparation of cDNA libraries were performed following the NovogeneAIT protocol. Sequencing was performed using a HiSeq platform with paired end 150 bp reads (PE150). The quality of raw data was assessed using FastQC. To analyze the sequence reads, the *S*. Typhi strain CT18 NC_003198 was used as a reference genome. Differentially regulated genes with more than twofold changes in expression in acid versus neutral pH with a q value of <0.005 were further analyzed by qRT-PCR. qRT-PCR: WT, *ompR*, *phoP*, and *ssrB* null strains were grown in LB medium overnight and sub-cultured in LB medium, pH_e_ 4.5, until the OD_600_ reached ~1.0. The total RNA was isolated using the RNeasy mini kit (Qiagen). After DNase treatment of the isolated RNA, the cDNA was synthesized using iScript Reverse Transcription Supermix (Biorad). Quantification of cDNA was carried out using SsoFast TM Eva Green Supermix (Biorad), and real-time amplification of the PCR products was performed using the iCycler iQ real-time detection system (Biorad). The mRNA expression level of the target gene was normalized relative to the 16S rRNA (*rrsA*) expression level. The primers used are listed in Table 2.

### Purification of OmpR for electrophoretic mobility shift assays (EMSAs)

OmpR was purified as previously described (38). EMSAs were performed using the LightShift Chemiluminescent EMSA Kit (Thermo Fisher #20148) according to the manufacturer’s instructions. The *cysK* promoter was amplified using primers listed in Table 2. Then, 10 fmol of biotinylated DNA was used in a 15 µL reaction containing binding buffer (10 mM Tris, pH 7.5, 50 mM KCl) along with 2.5% (wt/vol) glycerol, 1 mM MgCl_2_, 0.05% (wt/vol) Nonidet P-40, and 1 µg poly(dI-dC). To ensure the specificity of the reaction, a control with 100-fold excess of unlabeled DNA was performed. OmpR protein was added at the indicated concentrations, and samples were separated by electrophoresis on 5% non-denaturing acrylamide gels, run in 0.5× Tris-acetate buffer with EDTA.

### Infection of THP-1 macrophages

THP-1 (TIB-202) was purchased from the ATCC. THP-1 cells were routinely cultured in RPMI-1640 medium, supplemented with 10% fetal bovine serum (opti-gold, Gene Depot), 2 mM L-glutamine (Sigma), 1% modified Eagle’s medium amino acid solution (Gibco), 1 mM sodium pyruvate (Sigma), and 1% penicillin/streptomycin solution (Gene Depot) at 37°C with 5% CO_2_ and 80% humidity. For microscopy, phenol-free media were used. For infections, THP-1 cells were seeded in 24 (Corning) or 8 (Starsted) well plates for CFU or imaging, respectively, at a density of 1 × 10^5^ cells per well. Cells were differentiated into macrophage-like cells by treatment with 10 µM of phorbol 12-myristate 13-acetate (PMA; P1585; Sigma) for 48 h. A single colony from a fresh plate of *S*. Typhi was used to inoculate 3 mL of LB, and the culture was inoculated overnight (~16 h) at 37°C, standing conditions, to an OD_600_ of ~0.6. Bacterial cultures were used to infect differentiated THP-1 cells at a multiplicity of infection (MOI) of 10. The plates were centrifuged for 5 min at 600 × *g* to synchronize bacterial uptake, and the infection was performed for 25 min. THP-1 cells were then washed with Dulbecco’s phosphate buffered saline (DPBS) containing 100 µg/mL gentamicin and incubated in RMPI-1640 medium containing 100 µg/mL gentamicin for 30 min. The media were then replaced by RMPI-1640 containing 12 µg/mL gentamicin for the remainder of the experiment (this was time 0, T0). Alternatively, THP-1 cells were lysed to determine bacterial uptake. For enumeration of colony-forming units (CFUs), THP-1 cells were lysed by addition of Triton X-100 0.1% (vol/vol) in DPBS for 10 min. Intracellular bacteria were enumerated by serial dilution on agar plates. Four replicates were created for each time point. For microscopy, THP-1 cells were seeded into glass bottom eight-well chambers (Starsted AG), and phenol-free media were used. If required, the cells were fixed at the stated time point using 3% paraformaldehyde for 10 min at RT.

### Confocal imaging of promoter activity and redox state

All imaging was performed using the Leica DMI-8 scanning confocal system. For *in vitro* imaging, *Salmonella* harboring P*cysK*-mCherry, P*sifA*-mTagBFP2 fusions, or pFPV25::roGFP2 was grown overnight and sub-cultured in LB medium at pH_e_ 7 or 4.5 until the OD_600_ reached ~1.0. For arabinose-inducible SsrB, induction was at OD_600_ = 0.5 with 0.2% arabinose for 3 h. Bacterial cells were placed onto microscope slides (Toshinriko) coated with 1.5% (wt/vol) ultra-pure agarose and covered with a high-resolution glass coverslip (Carl Zeiss). For measuring P*cysK*-mCherry activity in *vitro*, the mCherry fluorescence emission at 611–650 nm was collected with an HyD detector after excitation at 587 nm. Alternatively, infected THP-1 cells in glass bottom chambers were analyzed at each time point mentioned. To create a mask for mCherry intensity measurements and to detect bacteria in THP-1 cells, the GFP emission at 500–550 nm was recorded after excitation at 488 nm. For P*sifA*-mTagBFP2 measurements, two sets of fluorescence data were collected corresponding to mTagBFP2 (excitation: 405 nm/emission 440–470 nm, PMT detector) and mCherry (excitation 561 nm, emission 600–630 nm, HyD detector). The P*sifA* activity recorded corresponded to the fluorescence intensity of mTagBFP2 divided by the constitutively expressed mCherry fluorescence.

For measuring the redox state in bacteria using roGFP2 (39), *in vitro* samples or infected THP-1 cells were incubated with 20 or 50 mM N-ethylmaleimide (NEM, Sigma) for 5 min and fixed with 1.5 or 3% PFA, respectively, and washed with PBS. The emission at 500–550 nm with an HyD detector was recorded after excitation at 405 and 480 nm. The bacterial redox state is described by the 405/480 nm ratio for each individual bacterium. Ratios were normalized to the maximum reduced and oxidized controls using the following formula: 0.9 × [(Ratio-MaxRed)/(Max Ox-Max Red)] + 0.1, as described in reference 43, where ratio is the fluorescence intensity value at 405/488 nm, Max Red and Max Ox correspond to the maximum reduced and maximum oxidized values, respectively. The maximum reduced and oxidized values were obtained by incubating control samples with 40 mM DTT or 100 mM H_2_O_2_, respectively. Z stacks were acquired at steps of 0.3 µm.

### Image analysis

All image analysis was performed with ImageJ 1.53C. Ratios were determined with the RatioPlus plugin. The background autofluorescence was subtracted during analysis.

## RESULTS

### Identification of *S*. Typhi genes that were regulated in response to acid stress

**Fig 1:**
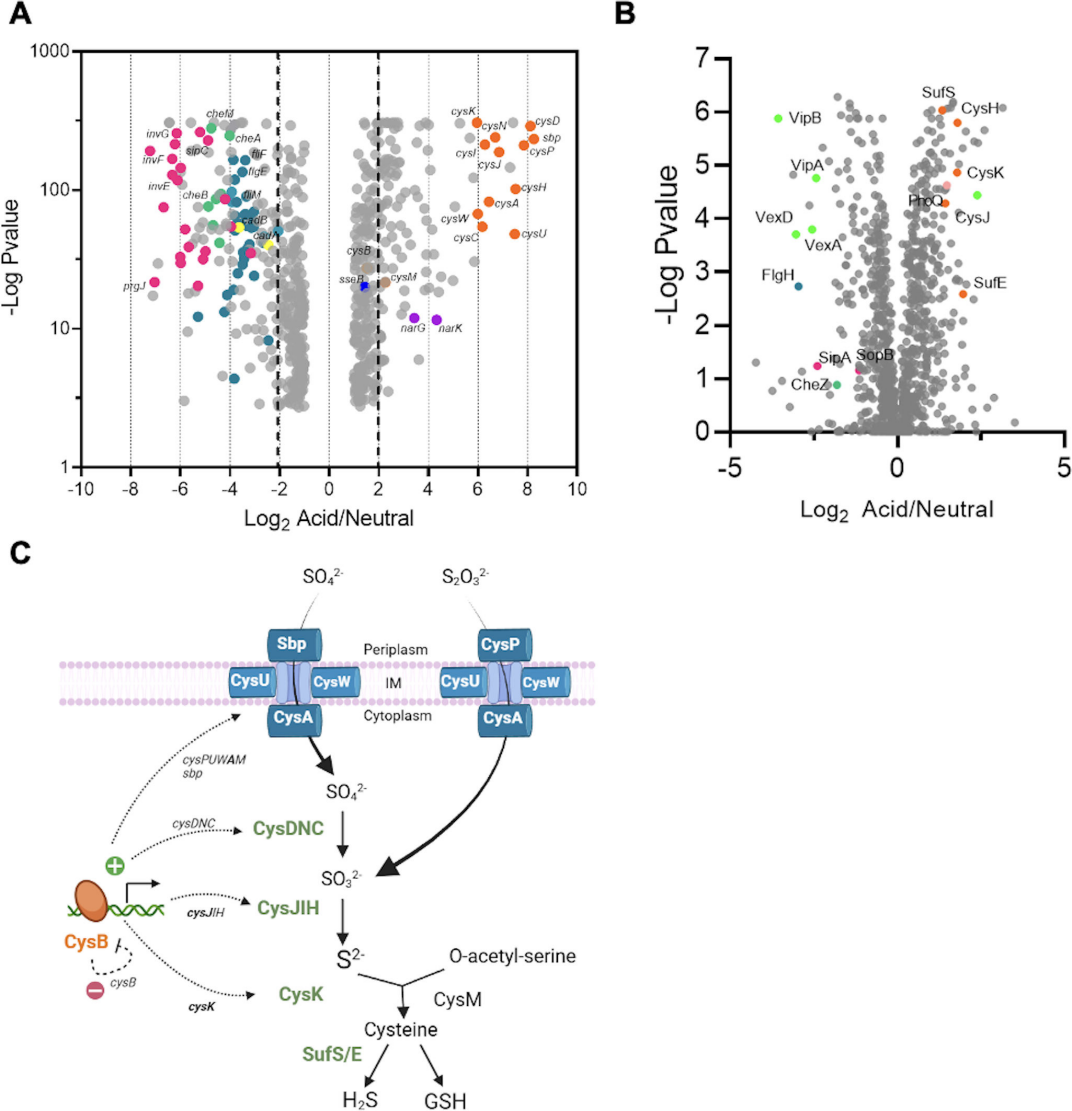
Analysis of *S*. Typhi identifies an upregulation of the sulfur assimilation pathway in response to acid pH. *Salmonella* Typhi was grown in LB at neutral (pH_e_ = 7) or acidic pH (pH_e_ = 4.5), and cells were harvested for RNAseq (**A**) or mass spectrometry analysis (**B**). The data were analyzed and plotted as fold change of the acidic condition versus the neutral condition. The left side of both panels indicates downregulated in acid pH, whereas the right side is upregulated in acid pH. The genes are color-coded by categories (purple: nitrogen pathway; dark blue: SPI-2; magenta: SPI-1; light blue: flagella; light green: capsule; dark green: chemotaxis; orange: cysteine pathway. (**C**). The sulfur assimilation pathway in *Salmonella*.

**Fig 2:**
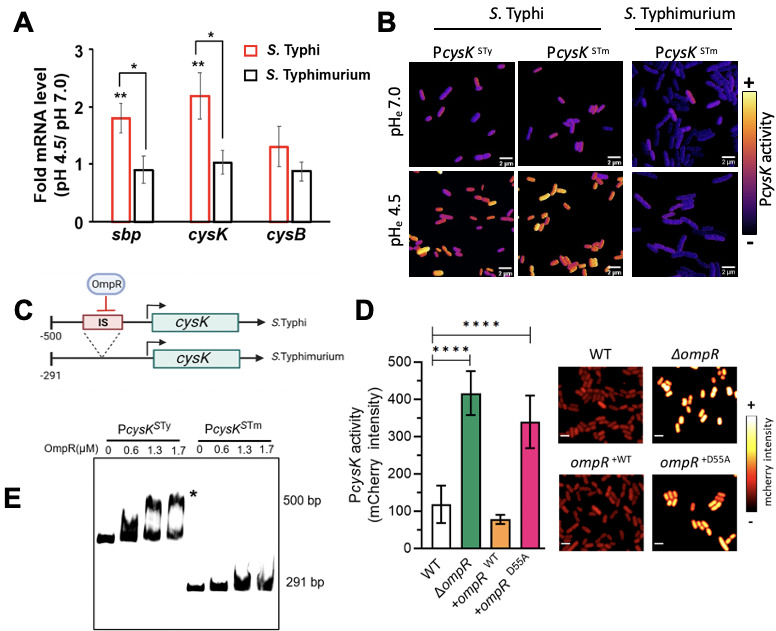
Regulation of sulfur metabolism differs in STy and STm. (A) *Salmonella* strains were grown in LB at neutral (pH_e_ = 7) or acid pH (pH_e_ = 4.5) to late exponential phase, and cells were harvested for RNA extraction and subsequent qRT-PCR (see Materials and Methods). The mRNA levels of *sbp, cysK,* and *cysB* at pH_e_ 4.5 were compared with pH_e_ 7.0 in the WT *S*. Typhi (red bars) and *S*. Typhimurium strains (black bars). The mRNA expression levels of the target genes were normalized relative to 16S rRNA. The error bars represent the mean ± standard deviation (*n* = 3). **P < 0.005, *P < 0.05, Student’s *t*-test. (B) *Salmonella* strains expressing a P*cysK*-mCherry of *S*. Typhimurium (P*cysK*^STm^) or S. Typhi (P*cysK*^STy^) compared with constitutively expressed ceruleans. Bacteria were grown at neutral (pH_e_ = 7) or acid pH (pH_e_ = 4.5). P*cysK* activity was obtained as the ratio of mCherry/ceruleans fluorescence. Representative images from three independent experiments are shown. (C) P*cysK* of *S*. Typhi includes an insertion sequence (IS, Top panel). Note the scales differ due to the 209 bp IS. (D) In the absence of *ompR,* P*cysK* activity is increased (green column) compared with the WT (white column). Complementation with *ompR* in *trans* decreases P*cysK* activity (orange). Repression is abolished when the *ompR* null strain is complemented with a substitution (D55A) that eliminates phosphorylation (magenta). Bacteria were grown in LB at pH_e_ 7.0 and harvested for confocal analysis. P*cysK* activity was obtained by direct measurement of mCherry fluorescence ****P < 0.0001, Student’s *t*-test. (E) OmpR represses P*cysK* by direct binding. Electrophoretic mobility shift assays were performed with purified OmpR protein incubated with P*cysK* from *S*. Typhi (left) vs S. Typhimurium (right). OmpR does not bind to the P*cysK* from *S*. Typhimurium.

To identify genes that were affected by acid stress in *S*. Typhi, we carried out RNA-seq and mass spectrometry analysis at acid and neutral pH (Fig. 1A and B). Most notably, a set of genes related to the cysteine biosynthetic pathway was significantly induced and was among the genes that were most highly upregulated in acid pH. The cysteine biosynthesis pathway catalyzes sulfur assimilation from sulfate or thiosulfate to form cysteine (Fig. 1C). It is composed of *sbp* and the *cysPUWA* operon responsible for sulfur uptake, *cysDNC* and *cysJIH* responsible for sulfur assimilation, as well as *cysE* and *cysK* for L-cysteine biosynthesis. These genes are controlled in *E. coli* and *Salmonella* by the transcriptional regulator CysB (30). According to our RNA-seq results (Fig. 1A), 13 out of the 14 genes in this pathway were upregulated in acid pH. The *cysE* gene involved in cysteine biosynthesis was unchanged between acid and neutral pH (log_2_ acidic/neutral = −0.221). The *cysB* gene presented a slight increase that was not significant (log_2_ acidic/neutral = 1.51). Mass spectrometry also confirmed that CysK and CysJ were similarly upregulated in acid pH (Fig. 1B), confirming our RNAseq results. In addition, proteomics analysis revealed that SufS and SufE, responsible for cysteine desulfuration, and H_2_S synthesis pathways were also upregulated in acid pH. To validate these results, we performed qRT-PCR on selective targets in acid and neutral pH (Fig. 2A). The qRT-PCR results were consistent with our RNAseq analysis, indicating a significant increase in *sbp* and *cysA* in acid pH. The transcription of *cysB* was not significantly affected by acid pH, as we observed by RNAseq (Fig. 1A). Taken together, these results highlight that activation of the sulfur assimilation pathway is an important response of *S*. Typhi to acidic stress.

In addition to cysteine biosynthetic genes, RNAseq highlighted additional genes that were upregulated in acid stress (log_2_ acidic/neutral > 3). These included a cystine transporter *tcyP*, spermidine synthesis genes *speD* and *speE*, genes involved in pyrimidine metabolism (*pyrE, pyrB, pyrI, carA*, and *carB*), the uracil transporter *uraA*, the cytosine permease *codB*, genes involved in anaerobic respiration by nitrate (*narK, narG,* and *napF)*, heat shock response genes *ibpA* and *ibpB*, DNA damage (*priB*), ribosomal subunits (*rplBCD, rplP, rplS, rplS, rplV, rplW, rpmC, rpsC,* and *rpsJ*), a glycyl radical cofactor (*grcA*), citrate lyase (*citC*), D-ribose transporter (*rbsD*), and six genes of unknown function. Some of these genes have been reported previously in the OmpR-dependent acid/osmotic stress response of *Salmonella* (17).

Finally, to our surprise, SPI-2-related genes were not significantly upregulated in acid stress, as was previously reported for *S*. Typhimurium (23, 44–46). Only *sseB* (encoding a component of the translocon) was slightly up (log_2_ acidic/neutral = 1.5), but in our RNAseq analysis, it was not significant (see Discussion). However, both RNAseq and mass spectrometry analysis highlighted a substantial downregulation of SPI-1 genes, flagellar-related genes, as well as chemotaxis-associated genes in acid pH, consistent with previous observations in *S*. Typhimurium (45, 47).

### Regulation of sulfur metabolism differs in *S*. Typhi and *S*. Typhimurium

Although *S*. Typhi and *S*. Typhimurium share 89% of genes (48), and previous results from our laboratory and others have demonstrated a substantial divergence in gene regulation in different lifestyles, such as biofilm formation between the two serovars (4, 49). It was therefore worthwhile to analyze the regulation of sulfur metabolism in response to acid pH in *S*. Typhimurium using qRT-PCR. Intriguingly, unlike what we observed with S. Typhi, neither *sbp* nor *cysK* was upregulated under acidic conditions in *S*. Typhimurium (Fig. 2A). Specific acid induction of *cysK* in *S*. Typhi was further validated by examining the activity of the *cysK* promoter (P*_cysK_*) using a transcriptional fusion to mCherry in both *S*. Typhi and *S*. Typhimurium. In *S*. Typhi, P*cysK* activity was increased about twofold in acid pH compared with neutral pH (Fig. 2B; Fig. S1A), and similar results were obtained with S. Typhi Ty2 (data not shown). In contrast, P*cysK* in *S*. Typhimurium 14028s was similarly low at neutral and acid pH, corroborating our qRT-PCR results (Fig. 2A). To determine whether the lack of a requirement for CysK in Typhimurium was more generalizable, we tested *S*. Typhimurium 20081, an invasive strain of the ST34 lineage (50). In 20081, CysK was expressed at neutral pH (unlike 14028), but was not required at acid pH (Fig. S2A).

To validate the significance of the cysteine pathway upregulation under acid stress, we next determined whether the pathway was upregulated during cell infections of human THP-1 macrophages. We first measured the expression of P*cysK*^STy^ upon infection of THP-1 cells by *S*. Typhi. The fluorescence intensity of the P*cysK*-mCherry construct revealed an activation of the *cysK* gene upon entry of *S*. Typhi within THP-1 cells, with a maximum expression at 6 h post-infection (hpi) that was ~3-fold greater than in the initial inoculum (Fig. S3), which then declined slightly (to ~2.5-fold) over the next 18 h. These results were in agreement with previously published transcriptome analysis, which reported a biphasic expression of P*cysK* (49).

### OmpR~*P* represses *cysK* in S. Typhi

Genes of the cysteine biosynthesis pathway are highly conserved in bacteria and have been well studied in *E. coli* and *Salmonella* (30). Half of the promoters are conserved between the two genera. A sequence alignment of P*cysK*^STm^ and P*cysK*^STy^ highlighted a significant difference between the *Salmonella* serovars. In *S*. Typhi, there was a 200 bp insertion sequence flanked by repeated sequences (Fig. 2C). To investigate the role of the insertion sequence in the acid stress response, we expressed P*cysK*^STm^-mCherry in *S*. Typhi and measured the promoter activity *in vitro* in either neutral or acidic conditions. In an *S*. Typhi background, the P*cysK*^STm^ was induced in acid pH (Fig. 2B), highlighting the importance of the H58 *S*. Typhi background in the induction of *cys* genes during acid stress.

We next investigated the effect of response regulators that were known to be involved in gene regulation during acid stress, i.e., OmpR, PhoP, and SsrB (17–19, 23, 51) (Fig. 2D and E; Fig. S1 and S4). In the wild-type (WT) strain, the activity of P*cysK* was low (~100 arbitrary fluorescence units). In the absence of *ompR,* P*cysK* activity increased fourfold, suggesting that OmpR was functioning as a repressor in *S*. Typhi (Fig. 2D). A substitution of OmpR that eliminated the phosphorylation site (D55A) exhibited similar activity as the null strain, indicating that phosphorylation of OmpR was required for its effect in downregulating P*cysK*. These results were corroborated using qRT-PCR (Fig. S4). Further analysis revealed that OmpR bound to P*cysK*^STy^ by direct binding to the insertion sequence specifically located in the *S*. Typhi *cysK* promoter (Fig. 2E; Fig. S1). Taken together, these results demonstrated a specific acid induction of the *S*. Typhi cysteine pathway that involved a role for OmpR~P, as well as additional unknown regulatory mechanisms that were specific to the *S*. Typhi serovar and not observed in *S*. Typhimurium 14028s (see Discussion).

### Sulfur metabolism protects *S*. Typhi against excessive oxidative stress and promotes its survival in THP-1 cells

**Fig 3:**
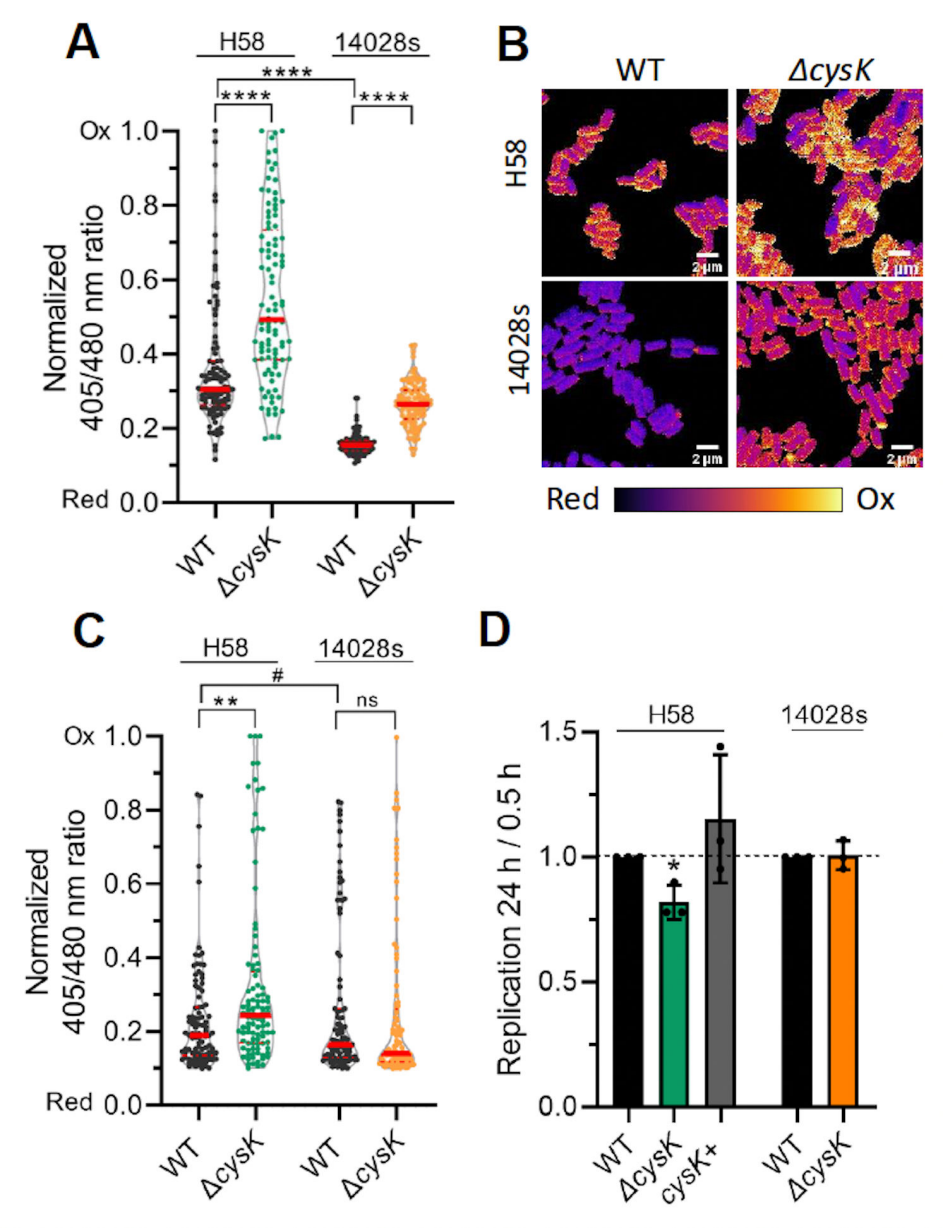
*S*. Typhi experiences higher redox stress compared with *S*. Typhimurium. (**A, B**) *Salmonella* strains expressing RoGFP_2_ were grown under acidic (pH_e_ = 4.5, SPI-2-inducing) conditions or (**C**) were used to infect THP-1 cells and then analyzed after 24 hpi. Cells were incubated with NEM and fixed as described in Materials and Methods and then analyzed by confocal microscopy. The emission at 550 nm was collected (excitation = 405 and 480 nm) for each individual bacterium, and the 405/480 ratio was plotted. (A and C) A total of 50 bacteria from two separate experiments were analyzed for each sample. **P < 0.001, ****P < 0.0001, Student’s *t*-test, the red bar is the median. The results were normalized according to the maximum oxidized and reduced values (see Materials and Methods). (**B**) Representative images *in vitro* are color coded for the redox state. Scale bar is 2 µm. (**D**) Deletion of *cysK* results in a bacterial replication defect in THP-1 cells for *S*. Typhi (green column) but not for *S*. Typhimurium (orange). After infection, THP-1 cells were lysed, and the intracellular bacterial load was determined by agar plating. Bacterial replication between 0.5 and 24 hpi was plotted relative to the corresponding WT strain. *P < 0.01, Student’s *t*-test.

The cysteine pathways and their derived compounds such as H_2_S or glutathione are associated with redox homeostasis in bacteria and are thus tightly regulated (52). We were interested in determining how the *cysK* mutant might influence the bacterial redox state compared with the WT in *S*. Typhi and *S*. Typhimurium strains. To examine this, we employed the redox-sensitive roGFP2 probe to monitor the bacterial redox state (43) both *in vitro* and during host cell infection. The roGFP2 is a substituted GFP that contains two cysteines that create a disulfide bond during oxidation, which interferes with its fluorescence. Using this probe, we detected that *S*. Typhi experiences significantly higher (>3-fold) oxidative stress than *S*. Typhimurium 14028s when bacteria were grown under SPI-2-inducing acidic conditions (Fig. 3A and B). *S*. Typhi H58 is also more highly oxidized than invasive *S*. Typhimurium 20081 (Fig. S4B). Furthermore, in *S*. Typhi, the response was highly heterogeneous compared with *S*. Typhimurium. This difference in redox stress between *S*. Typhi and *S*. Typhimurium under acidic conditions likely results in differential gene expression between these two serovars and might explain the specific induction of the cysteine pathways in *S*. Typhi. We next compared the effect of *cysK* deletion on the redox state of *S*. Typhi and *S*. Typhimurium. Deletion of *cysK* resulted in an increase in the oxidative state of both serovars (Fig. 3A and B). However, this increase was moderate in the case of *S*. Typhimurium, where the redox state of the *cysK* mutant did not even reach the oxidized level of the WT *S*. Typhi (compare black circles of each serovar). In contrast, the increase in oxidation in the *S*. Typhi *cysK* mutant was substantial (a normalized median ratio of ~0.5), with some bacteria fully oxidized, whereas the normalized median ratio of the *cysK* mutant in *S*. Typhimurium was <0.3 (compare green and orange circles).

We next investigated the redox balance of *Salmonella* strains in the context of infection in THP-1 macrophages (Fig. 3C). Overall, at 24 hpi, both serovars demonstrated substantial heterogeneity, although *S*. Typhi was more oxidized than *S*. Typhimurium. Deletion of *cysK* in *S*. Typhi leads to a general increase in the redox state towards a more oxidized level, as we observed *in vitro* (Fig. 3A). Similarly, deletion of *cysK* in *S*. Typhimurium had little effect on the redox state (Fig. 3C). This result emphasizes a heretofore unidentified feature of *S*. Typhi, which experiences higher redox stress and is distinct from *S*. Typhimurium in requiring the sulfur assimilation pathway to maintain redox homeostasis upon acid stress or growth *in vivo* (see Discussion).

As *S*. Typhi experienced excessive redox stress in THP-1 cells (Fig. 3A through C), we next investigated its intracellular survival, using a gentamicin protection assay and bacterial enumeration (Fig. 3D). In *S*. Typhi, CysK was also important for intracellular replication because the replication rate of the *cysK* null mutant was reduced to ~80% compared with the WT strain (Fig. 3D, left). Complementation of *cysK* in *trans* restored bacterial replication to WT levels (gray bars). Again, *cysK* deletion had no effect on *S*. Typhimurium replication; the null strain was identical to the WT (Fig. 3D, right, Fig. S5A). These results were well correlated with the redox results and highlighted a central role for sulfur metabolism in *S*. Typhi pathogenesis during host cell infection.

### Sulfur metabolism influences SPI-2 expression in *S*. Typhi

**Fig 4:**
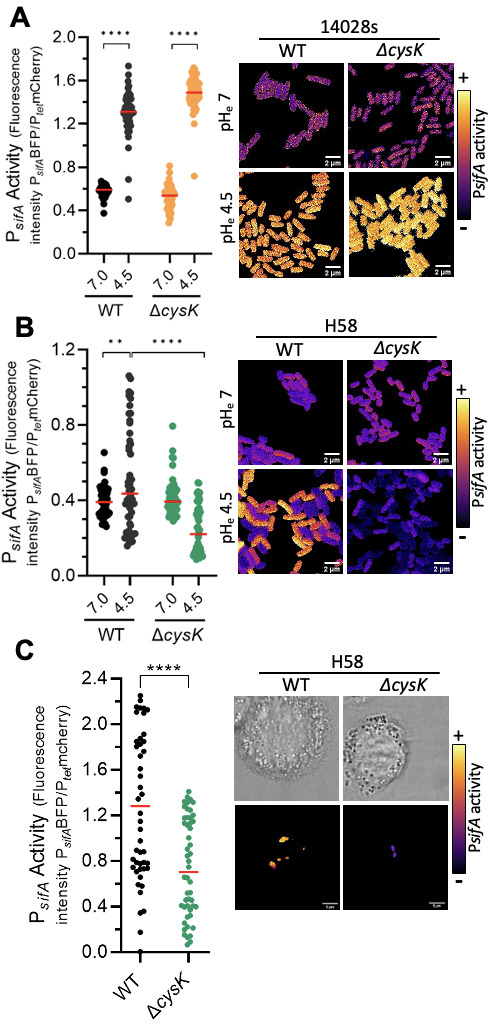
SPI-2 is downregulated in an *S*. Typhi *cysK* null strain. SPI-2 activity was analyzed in *S*. Typhi (**A**) or S. Typhimurium (**B**) using the transcriptional fusion P*sifA-mTagBFP*, expressed from a plasmid that also contained a constitutively expressed P*Tet*-mCherry fusion. In *S*. Typhi H58, *sifA* expression was stimulated by acid stress (black circles, left). In the *cysK* null mutant, the activity of P*sifA* actually decreased in acid pH compared with neutral pH (green circles). In *S*. Typhimurium 14028, there was essentially no difference in P*sifA* activity between the WT and the *cysK* null strain. Bacteria were analyzed by confocal microscopy for fluorescence emission from P*sifA-mTagBFP* and P*Tet*-mCherry. The fluorescence from mTagBFP was divided by the mCherry fluorescence for each individual bacterium to determine the P*sifA* activity. Representative images are shown. One dot represents one bacterium; 50 bacteria were analyzed with the median shown as a red bar. **P < 0.05, ****P < 0.0001, Student’s *t*-test (right panel). Representative images were color-coded for P*sifA* activity (left panel). Scale bar, 2 µm. (C) Expression of SPI-2 was monitored in infected THP-1 cells at 24 hpi. Infected THP-1 cells were imaged by confocal microscopy, and mCherry and mTagBFP2 were recorded for each individual bacterium (*n* = 50). ****P < 0.0001, Student’s *t*-test. Representative images are shown; the scale bar is 5 µm.

The redox state in bacteria affects gene expression. Recent studies, as well as our unpublished results, established a role for SPI-2-related genes during *S*. Typhi infection of THP-1 cells (53; M. Fernandez, S. Deolankar, and L. J. Kenney, unpublished data). Since our results of S. Typhi invasion suggested that the cysteine pathway played a role in bacterial survival in THP-1 cells (Fig. 3). It was then worthwhile to investigate the relationship between the sulfur assimilation pathway and SPI-2-related gene expression. The *sifA* gene is under the control of SsrB and is strongly induced by acid pH (23, 54). Its activity was monitored using a P*_sifA_*-BFP reporter construct under acid-inducing conditions (Fig. 4). The fluorescence intensity of P*_sifA_*-BFP increased >2-fold upon exposure to acid pH, and the response was homogenous in *S*. Typhimurium (Fig. 4A). In contrast, P*sifA* induction in *S*. Typhi was less robust (~1.4-fold), and much more heterogeneous. This may in part explain the lack of SPI-2-related genes in our RNAseq analysis. The induction of P*sifA* was completely abolished in the *cysK* null mutant of *S*. Typhi (Fig. 4B), but deletion of *cysK* in *S*. Typhimurium had no effect on *sifA* expression (Fig. 4A). These *in vitro* effects were also observed during infection of THP-1 cells, where deletion *of cysK* in *S*. Typhi prevented P*sifA* activity at 24 hpi (Fig. 4C). Deletion of *cysK* did not affect P*sifA* activity in the *S*. Typhimurium 14028s strain (Fig. S1B).

### SsrB function is impaired in a *cysK* null background and relieved by a Cys203 mutant

**Fig 5:**
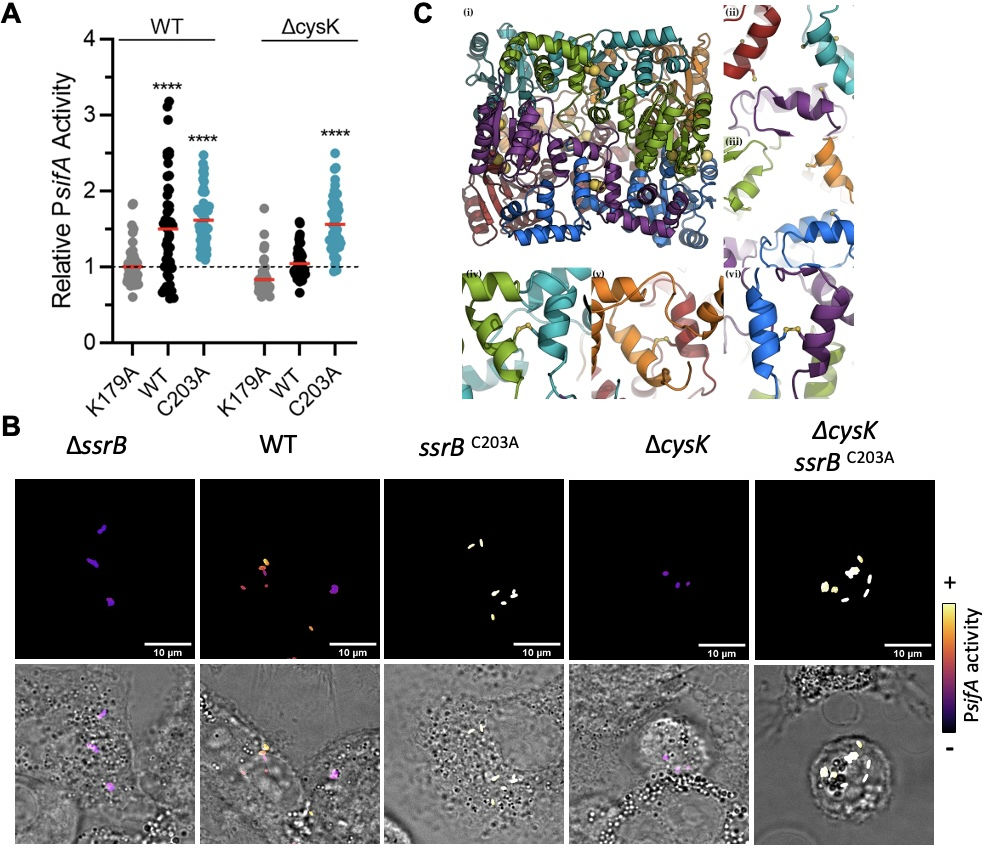
Cysteine mutants of SsrB restore *sifA* activation in THP-1 macrophages. (A) SsrB variants were expressed after arabinose induction in H58 strains expressing or lacking *cysK*. The K179A variant of SsrB was used as a negative control. SPI-2 activity was analyzed in *S*. Typhi H58 after growth in LB pH 4.5 using the transcriptional fusion P*sifA-mTagBFP2* that also contained a constitutively-expressed P*Tet*-mCherry fusion. The fluorescence from mTagBFP2 was divided by the mCherry fluorescence for each individual bacterium to determine the P*sifA* activity. The results were normalized to the fluorescence average of the H58 WT strain expressing SsrB K179A. One dot represents one bacterium; 50 bacteria were analyzed with the median shown as a red bar. ****P < 0.0001 by ANOVA test. (B) A C203A mutant of SsrB restores P*sifA* activity in a *cysK* null background. THP-1 macrophages were infected with *S*. Typhi H58. At 24 hpi, cells were imaged by confocal microscopy, and mCherry and mTagBFP2 fluorescence intensities were recorded for each individual bacterial cell expressing the transcriptional fusion P*sifA-mTagBFP* from a plasmid, also containing a constitutively expressed P*Tet*-mCherry fusion. The fluorescence from mTagBFP was divided by the mCherry fluorescence for each individual cell to determine the P*sifA* activity. Representative images (out of 50) are shown, color-coded for P*sifA* activity. Scale bar, 10 µm. (C) The SsrB hexamer model after 10 ns of Molecular Dynamics. (i) The SsrB hexamer, the cysteine Sg atoms are highlighted as large yellow spheres. The disulfide bond, between dimers (Cys203_A and Cys203_B, etc.), was visible along the vertical centerline of the hexamer. This inter-chain disulfide is internal to the three SsrB dimers that form the threefold symmetric SsrB hexamer. (ii) The threefold hexameric axis (left side) showing the large pore formed by cysteine 46 from chains “B,” “D,” and “F.” The partially hidden Cys46 residues are buried in a hydrophobic pocket that stabilizes the N-terminal domain. (iii) The threefold hexameric axis (right side) showing the large pore formed by Cys46 from chains “A,” “C,” and “E.” (iv, v, vi) View of the disulfide between Cys203 at the dimer interfaces from top to bottom of panel i: (iv) chains “A” and “B,” (v) chains “E” and “F,” (vi) chains “C” and “D.” Colors by chain: A: green; B: turquoise; C: blue; D: purple; E: orange; F: red.

Since SsrB is the SPI-2 master regulator, an obvious explanation for the reduced *sifA* expression in the *cysK* null strain (Fig. 4) was due to an oxidizing impact on SsrB function. Most notably, Cys203 in SsrB is in the dimerization helix in the C-terminal DNA binding domain (55); it is known to be S-nitrosylated during exposure to acidified nitrite in the phagosome (56); our unpublished observations). We therefore examined the *sifA* activity in a *cysK* null strain, comparing activation of *sifA* in LB pH_e_ 4.5, by SsrB WT, or a mutant in which Cys203 was substituted with alanine (C203A). A substitution in the recognition helix that eliminated DNA binding (K179A) served as a negative control (Fig. 5A). In the H58 WT background, P*sifA* was activated upon induction of SsrB WT, whereas in the *cysK* null strain, the activity was similar to the negative control (Fig. 5A, black dots). P*sifA* activity was only restored in ∆*cysK* by induction of SsrB C203A, demonstrating that the *cysK* mutant encounters a high oxidation state that prevents SPI-2 induction (Fig. 5A, right). In parallel, we noted that overall, in an H58 background, P*sifA* activity was higher upon expression of SsrB C203A compared with SsrB WT (Fig. 5A). This result indicates that in LB pH_e_ = 4.5, a segment of the H58 population suffers from an oxidative state that is too high for successful SPI-2 activation. We next introduced the C203A substitution at the native *ssrB* locus to follow *sifA* activity in THP-1 at 24 hpi (Fig. 5B)**.** Similar to our *in vitro* observations, the C203A substitution restored P*sifA* activity in the *cysK* null strain, and it also increased the overall activation of *sifA* in H58. Taken together, these results demonstrate that H58 encounters oxidative stress in human macrophages that reduces SPI-2 activation, and this oxidative stress is moderated by the activation of *cysK*.

## DISCUSSION

### *S*. Typhi H58 is unique in its response to acid stress compared to *S*. Typhimurium

Although *S*. Typhimurium is often used as a surrogate for *S*. Typhi, our results highlight important differences as in the way that the two serovars respond to environmental stress, including the host environment. Our RNAseq and proteomics results identified the cysteine biosynthesis and sulfur assimilation pathways as being significantly upregulated in response to acid stress in *S*. Typhi H58 but not in *S*. Typhimurium (Fig. 1 and 2). Deletion of *cysK* resulted in a growth defect in H58-infected THP-1 macrophages, but *cysK* deletion had no effect on *S*. Typhimurium replication (Fig. 3). Furthermore, there were substantial differences in the regulation of *cysK*, the terminal gene in the cysteine biosynthetic pathway. In the case of H58, the *cysK* promoter contained an insertion sequence, and the global regulator OmpR bound to the upstream regulatory region but not to the *cysK* promoter of *S*. Typhimurium. OmpR acted to repress *cysK*, and repression required the phosphorylation site, Asp55 (Fig. 2; Fig. S2 and S3). In acid pH, OmpR~P levels would decrease, as the acyl phosphate intermediate is acid-sensitive (24), releasing the repression of *cysK* in the acidic vacuolar environment. This OmpR repression was not the basis for the differential regulation between *S*. Typhi and *S*. Typhimurium because when *PcysK*^STm^ was expressed in H58, the promoter was acid-induced, but not when it was in its native STm background (Fig. 2).

### Redox differences between H58 *S*. Typhi and *S*. Typhimurium

Our measurements of the oxidation state of H58 indicated that it was substantially more oxidized in response to acid pH both *in vitro* and *in vivo* compared with *S*. Typhimurium (Fig. 3). Deletion of *cysK* substantially increased the oxidation state of both serovars, but the effect was highly significant in H58, whereas the *cysK* null mutant of *S*. Typhimurium was similar to the oxidized state of the WT H58. Thus, the cysteine pathway is important for mitigating the oxidizing atmosphere that H58 encounters during exposure to acid pH in the SCV. Deletion of *cysK* reduced the ability of H58 to replicate in THP-1 macrophages, but had no effect on *S*. Typhimurium (Fig. 3). In keeping with our findings, a recent global analysis reported that deletion of *cysK* in the domesticated, antibiotic-sensitive *S*. Typhi Ty2 strain had a negative impact on bacterial growth in THP-1 cells (57). The decrease in replication was also evident as a reduction in the activation of SPI-2 effectors, as we observed a significant decrease in P*sifA* activity in the H58 *cysK* null strain. Again, deletion of *cysK* had no effect on *S*. Typhimurium with respect to *sifA* expression (Fig. 4).

The major transcriptional activator of SPI-2 genes is the response regulator SsrB. It undergoes a substantial conformational change in acid pH that is regulated by the amino acid His12 in the N-terminal receiver (phosphorylation) domain. The vacuolar drop in pH induces an His12-dependent oligomerization event (evidenced by a large increase in cooperativity, with a Hill coefficient of 12) that also increases its affinity for DNA by >30-fold by the C-terminus (23, 24). SsrB is unique in having vicinal sulfhydrals, with cysteine residues located at positions 45 and 46 in the receiver domain and a cysteine in the C-terminal dimer interface (C203) that have intrigued us for years (55). This arrangement is rather common in proteins (58), although the formation of vicinal disulfide bonds is rare (59–61). We constructed a homology model of an SsrB hexamer based on the crystal structure of RcsB formed at acid pH (62) in which the N-terminus of one monomer interacts with the C-terminus of an adjacent monomer (Fig. 5C). In the highly oxidizing environment that H58 encounters in the acidic vacuole, our data suggest that cysteines in SsrB formed disulfides that impaired function, which explains why SsrB-dependent genes, such as *sifA*, were downregulated in the *cysK* null strain (Fig. 4). Because the C203A mutant of SsrB could support *sifA* expression in the *cysK* null strain, we speculate that C203 likely forms an inactive disulfide during H58 oxidation. This finding links the oxidizing vacuole and changes in redox stress to structural changes in SsrB that impact SPI-2 expression in H58 and is supported by our observations that a cysteine mutant of SsrB restored *sifA* expression (Fig. 5). It is very likely that other post-translational modifications occur in this environment that would contribute to differences in gene regulation, and hence pathogenesis, in *S*. Typhi compared with *S*. Typhimurium.

What contributes to the increased oxidative stress of *S*. Typhi? In *S*. Typhimurium, two periplasmic superoxide dismutases (SodCI and SodCII) combat the action of the NADPH oxidase during the respiratory burst to promote *Salmonella* survival in the phagosome (63, 64). The *sodCI* gene is encoded on the lambdoid phage Gifsy2, which is not present in *S*. Typhi (65). Although it is still unclear exactly how the oxidative burst kills bacteria (66), it is tempting to speculate that the lack of *sodCI* might contribute to the increased oxidative stress experienced by H58 *in vitro* (Fig. 3A) and in THP-1 macrophages (Fig. 3C). Future studies will hopefully address this question and may provide an approach for combating *S*. Typhi infections.

## Data Availability

RNA-seq data are presented in Table S1.

## SUPPLEMENTAL MATERIAL

Supplemental Figures mbio.00467-25-s0001.docx Figures S1 to S5.

Table S1 mbio.00467-25-s0002.xlsx RNA-seq data.

ASM does not own the copyrights to Supplemental Material that may be linked to, or accessed through, an article. The authors have granted ASM a non-exclusive, world-wide license to publish the Supplemental Material files. Please contact the corresponding author directly for reuse.
