# Supplementary material for: The sulfur assimilation pathway mitigates redox stress from acidic pH in Salmonella Typhi H58
Source: mBio. 2025 May 27;16(7):e00467-25. doi: 10.1128/mbio.00467-25 (PMC12239559; doi:10.1128/mbio.00467-25)
Supplement: Supplemental Figures — Figures S1 to S5. [file mbio.00467-25-s0001.docx]

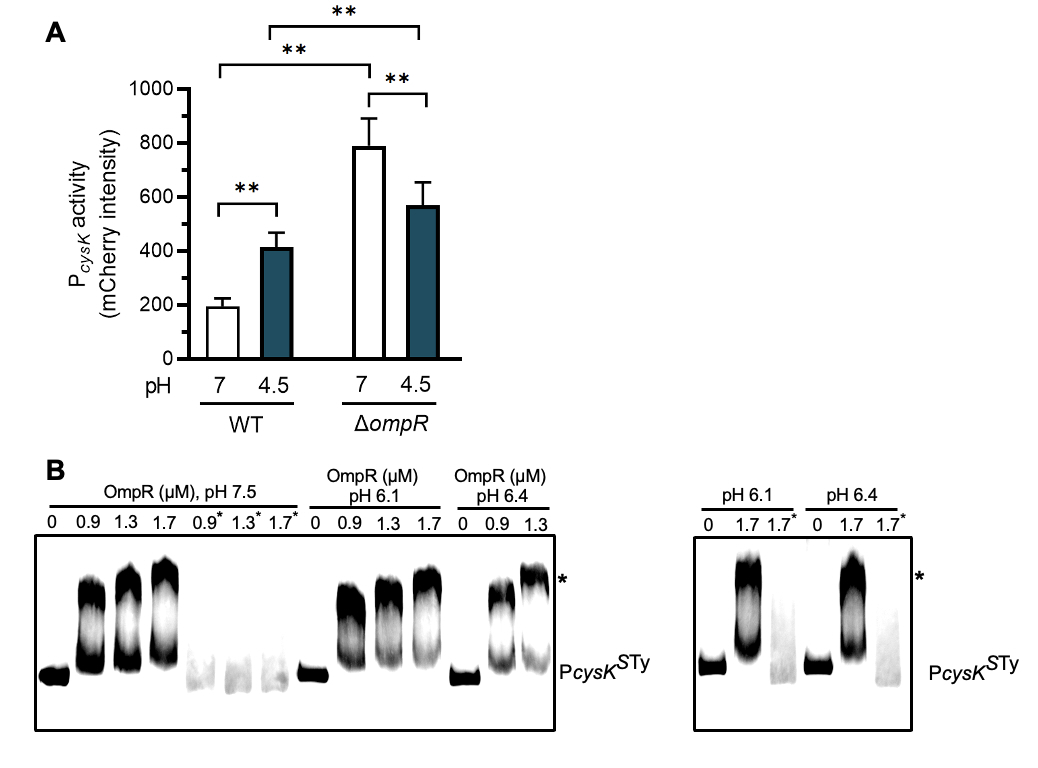


**Figure S1.** OmpR represses *S.* Typhi P*cysK* by direct binding*.* **A*.*** *S.* Typhi expressing a chromosomally-encoded P*cysK-*mCherry transcriptional fusion. Bacteria were grown in LB at pH_e_ = 4.5 or pH_e_ = 7.0 and cells were harvested for confocal microscopy. P*cysK* activity was obtained by direct measurement of the mCherry fluorescence. n = 50 bacteria. **p<0.05, Student’s T-test. **B**. Electrophoretic mobility shift assay with purified OmpR protein and P*cysK* from *S.* Typhi. *: 100x unlabeled DNA was added.


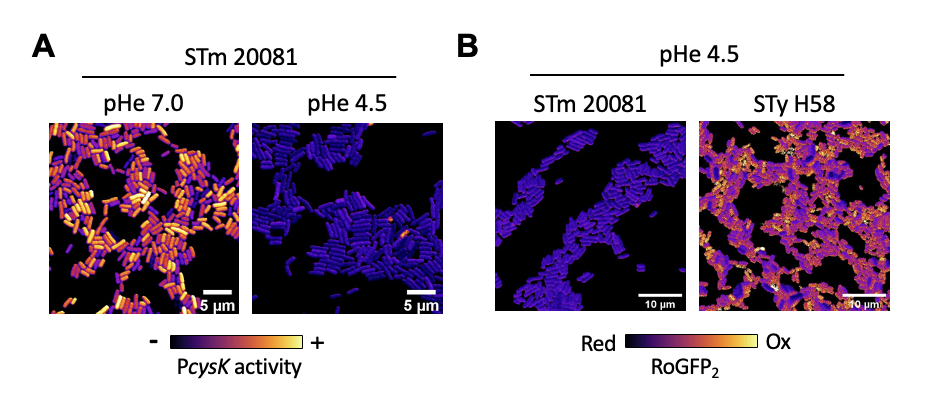


**Figure S2.** The acid response diverges between serovars Typhi and Typhimurium. **A.** *PcysK* is induced only in the acidic environment of serovar Typhi. *Salmonella* strains expressing a P*cysK*-mCherry of *S.* Typhimurium 20081 (P*cysK*^STm^) and constitutively expressed ceruleans were grown at neutral (pH_e_ = 7) or acid pH (pH_e_ = 4.5). P*cysK* activity was obtained as the ratio of mCherry/ceruleans fluorescence intensity, using the image J ratio plus plugin. Representative images are shown that are color coded for P*cysK* activity, the scale bar is 5 µm. In *S*. Typhimurium 20081, P*cysK* activity was high compared to *S*. Typhimurium 14028s (Figure 2), but activity was completely off in an acidic environment. **B.** *S.* Typhi experiences higher redox stress compared to *S.* Typhimurium serovars. *Salmonella* strains expressing RoGFP_2_ were grown under acidic conditions (pH_e_ 4.5, SPI-2-inducing). Cells were incubated with NEM, fixed, and then analyzed by confocal microscopy. The emission at 550 nm was collected (excitation = 405 and 480 nm) for each individual bacteria, and the 405/480 ratio was constructed using the image J ratio plus plugin. Representative images are shown. Scale bar is 10 μm.

**
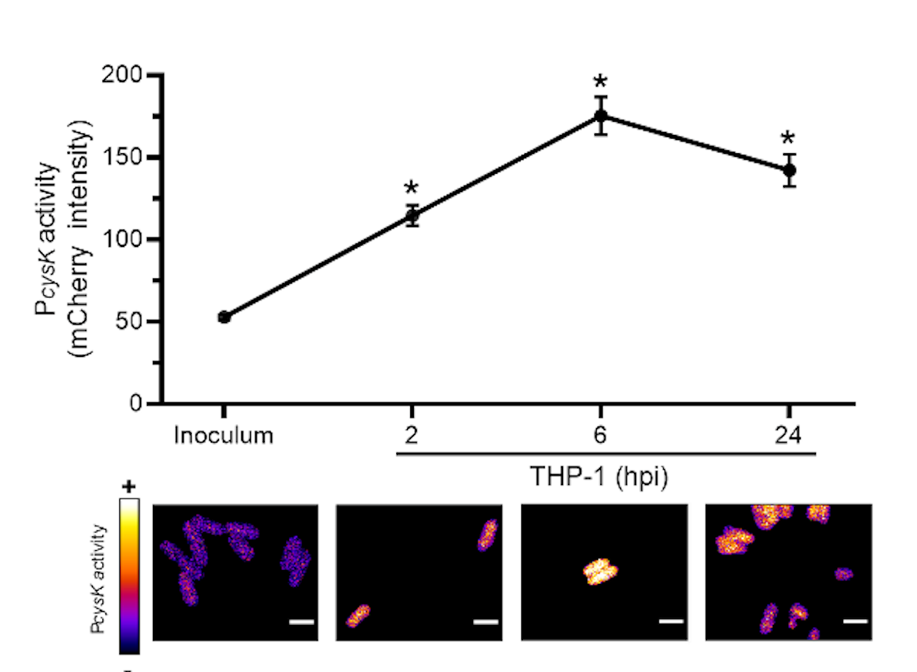
**

**Figure S3.** P*cysK* is activated upon THP-1 infection by *S.* Typhi. P*cysK* expression by *S*. Typhi in THP-1 cells peaks at 6 hpi and then decreases. THP-1 cells were infected with *S*. Typhi expressing a chromosomal P*cysK*-mCherry. The mCherry fluorescence intensity was recorded in 50 intracellular bacteria over time. The points represent the median of the mCherry intensity. Three independent experiments (150 bacteria total) were performed. Significant differences compared to the inoculum *p < 0.05, Student’s T-test. Representative images were color coded for P*cysK* activity at each time point. The scale bar is 2 μm.


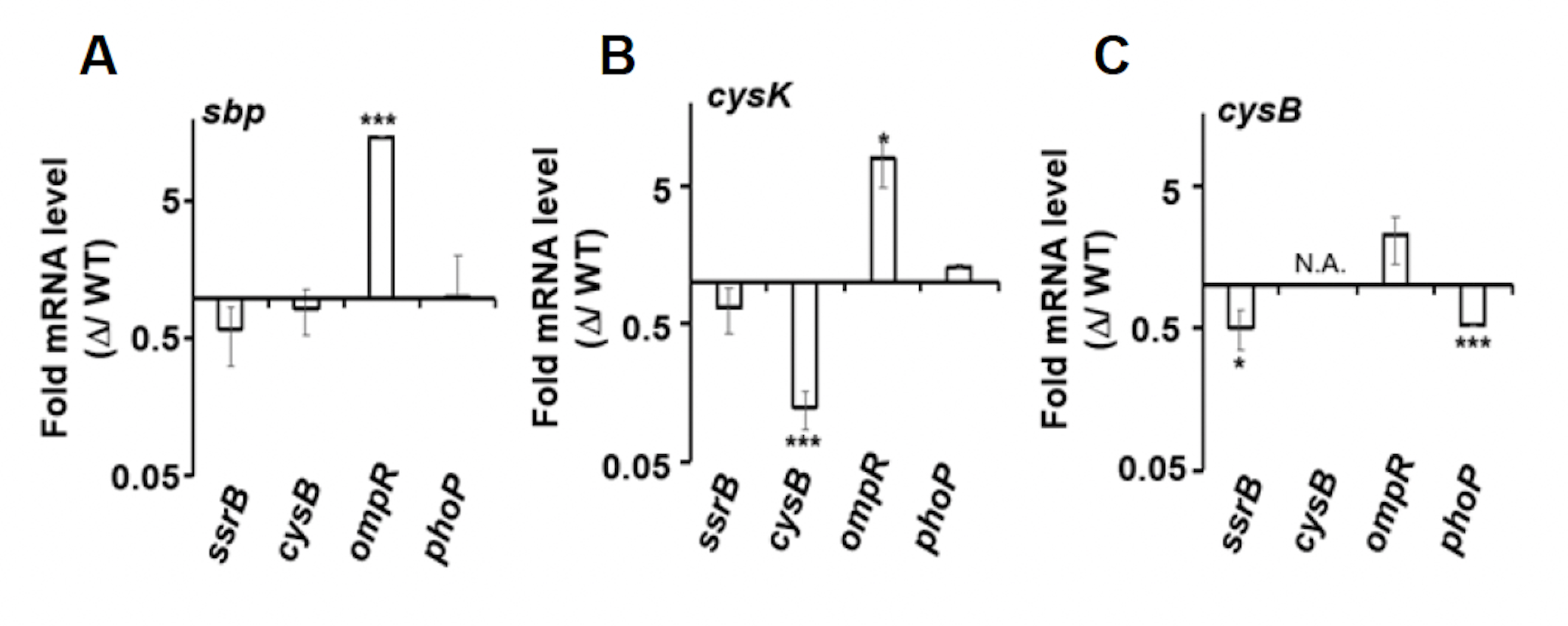


**Figure S4.** Transcriptional regulation of *sbp*, *cysK* and *cysB* by SsrB, CysB, OmpR and PhoP in *S.* Typhi. mRNA levels of *sbp* (**A**), *cysK* (**B**), and *cysB* (**C**) genes were determined by qRT-PCR from WT, *ssrB*, *cysB, ompR and phoP* null strains of *S.* Typhi grown at pH_e_ = 4.5. Fold expression changes in the null strains were compared to the WT level. The error bars represent the mean ± standard deviation (n = 3). *p<0.05, ***p<0.005, Student’s T-test.


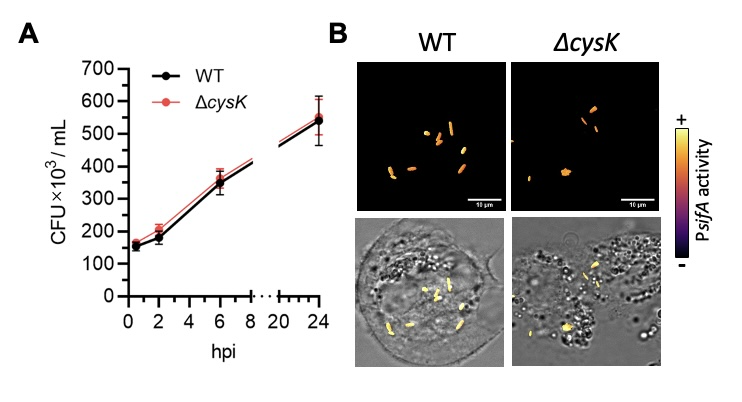


**Figure S5.** Deletion of *cysK* in *S.* Typhimurium does not impact virulence. **A**. Survival of *S.* Typhimurium 14028s in THP-1 cells is not affected by *cysK* deletion. After infection, THP-1 cells were lysed, and the intracellular bacterial load was determined by agar plating. The results represent one experiment of 3 independent experiments performed. **B**. SPI-2 activation by *S.* Typhimurium 14028s in THP-1 cells is unaffected by *cysK* deletion*.* Expression of SPI-2 was monitored in *S.* Typhimurium 14028s-infected THP-1 cells at 24 hpi. Infected THP-1 cells were imaged by confocal microscopy and mCherry and mTagBFP2 intensities were recorded for each individual bacteria expressing the transcriptional fusion P*sifA-mTagBFP* from a plasmid, which also contained a constitutively-expressed P*Tet*-mCherry fusion. The fluorescence from mTagBFP was divided by the mCherry fluorescence for each individual bacterial cell to determine the P*sifA* activity. Representative images (out of 50) are shown color-coded for P*sifA* activity, Scale bar = 10 µm.
